# Partial and complete microdeletions of Y chromosome in infertile males from South of Iran

**Published:** 2016-12

**Authors:** Raheleh Masoudi, Liusa Mazaheri-Asadi, Shahryar Khorasani

**Affiliations:** Department of Biology, College of Sciences, Shiraz University, Shiraz, Iran

**Keywords:** Microdeletions, Azoospermia, Infertility, Y chromosome

## Abstract

Y chromosome microdeletions are the second genetic cause of male infertility. The incidence of Y chromosome microdeletions can vary considerably depending on several factors, including patient selection criteria, population composition, and diagnostic protocols. They are associated with spermatogenic failure and lead to azoospermia or oligozoospermia. The advance in assisted reproductive technology and intracytoplasmic sperm injection, and the possibility of genetic defect transmission to the next generation make it necessary to improve our knowledge about the various factors leading to spermatogenic impairment. This study was designed to determine the frequency of microdeletions of Y chromosome in a population from South of Iran. 81 infertile males with non-obstructive azoospermia or oligozoospermia were selected. Multiplex PCR using several STS markers was carried out to detect the complete or partial microdeletions. The frequency of both complete and partial microdeletions in men with azoospermia or severe oligozoospermia was 7.4%. All microdeletions were observed in AZFc region. There was 1.25% complete microdeletions and after excluding complete microdeletions, we detected 5% gr/gr and 1.25% b2/b3 microdeletions. In our control group of fertile males, 4% gr/gr microdeletions was detected while there was no b2/b3 microdeletions. We concluded that there is a low frequency of Y chromosome microdeletions in a population of infertile males from South of Iran. b2/b3 microdeletions was detected only in infertile males and not in the control group. Screening a population with larger sample size is necessary to determine the involvement of this partial microdeletion in infertility of this population.

## INTRODUCTION

Infertility is the inability to conceive after one year of unprotected intercourse. In Iran, the prevalence of primary infertility is about 20%, which seems to be higher than the world average [1]. Male infertility accounts for 40-50% of the infertile cases [2]. Y chromosome microdeletions are the second most frequent genetic cause of male infertility [reviewed in 3]. Microdeletions occur in the long arm of Y chromosome (Yq) in a region known as azoospermia factor (AZF), including AZFa, AZFb, and AZFc [4]. Presence of palindromic sequences and homologous recombination lead to either partial or complete deletion of these regions [5, 6]. Removing of 792 kb of the Y chromosome and the only two genes of AZFa region occur in complete deletion of AZFa which results in complete sertoli cell only (SCO) syndrome and azoospermia. The frequency of this microdeletion is the lowest (0.5-4%) among the three regions [4, 7-10]. There are 24 genes with a total copy of 46 in AZFb and AZFc regions. Complete deletion of AZFb removes 6.2 Mb of the Y chromosome and 32 copies of genes [5]. This microdeletion leads to azoospermia. The most frequent complete microdeletion occurs in the AZFc which removes 3.5 Mb of the Y chromosome including 21 copies of genes [11]. The clinical phenotype of this microdeletion is variable from azoospermia to oligospermia. Partial deletions and duplications of AZFc also occur. Among several partial microdeletions, gr/gr deletion may have clinical relevance [11]. This microdeletion removes almost half of the AZFc gene content and leads to a range of spermatogenic phenotype from azoospermia to normospermia. Y chromosome microdeletions can lead not only to spermatogenesis failure, but also in recurrent pregnancy loss [12]. Moreover, there is always a risk of transmission of these microdeletions from father to son [13, 14]. Therefore, screening of these microdeletions is important especially when assisted reproductive technology (ART) is applied. The incidence of Y chromosome microdeletions is 2-10% or even higher among azoospermic patients with no sperm count or oligospermic patients with sperm count of less than 5 million per milliliter [3]. Depending on the patient selection criteria and population composition [3], the observed frequency of Y chromosome microdeletions may vary. Moreover, there are many diagnostic protocols which some are inaccurate [15, 16]. In addition, heterogeneity in the type and number of PCR markers applied in different researches may result in a range of frequencies of Y chromosome microdeletions observed in various investigations. In Iran, reports regarding the frequency of microdeletions vary considerably, as well. The highest prevalence (52%) of Y chromosome microdeletions was reported in azoospermic and oligospermic patients from North of Iran, Rasht [17]. The lowest frequency of microdeletions observed in Iran was 2.13% in Tehran [18]. This discrepancy can be due to experimental errors [15, 16] or various approaches applied to determine the frequencies. The aim of this study was to investigate the frequency of Y chromosome microdeletions in a population of infertile males from South of Iran using a standard method applied by European Academy of Andrology/European Molecular Genetics Quality Network (EAA/EMQN).

## MATERIALS AND METHODS

**Subjects: **In this study, 81 infertile males, including 53 non-obstructive azoospermic and 28 severe oligospermic patients (sperm count of less than 5 × 106) and 50 controls (proven fathers and/or normospermic male) were included. The average age was 35.8 in patients and 35 in controls. Patients attended to Dr Rostami's infertility center from November 2013 to December 2015. All patients were from South of Iran, including 66.7% Fars, 15.3% Tork, 14.1% Lor, 1.3% Kord, 1.3% Arab, and 1.3% others.

**Analysis of complete microdeletions: **After signing the consent form, blood samples were obtained and stored in -20°C. Boiling method was applied in order to extract the genomic DNA [19]. Concentration and purity of DNA was determined using Thermo Scientific TM Nanodrop spectrophotometer. Two sets of Multiplex PCR (A and B) (Table 1), suggested by EAA/EMQN, were applied to detect the complete microdeletions of AZFa, AZFb, AZFc regions [20]. STS primers used in these sets were sY84 and sY86 for AZFa, sY127 and sY134 for AZFb, and sY254 and sY255 for AZFc (Table 2).

**Table 1:** Multiplex A and B STS markers

**Multiplex A**	**Multiplex B**
sY14: 472 bp (SRY)	sY14: 472 bp (SRY)
sY86: 320 bp (AZFa)	sY84: 326 bp (AZFa)
sY127: 274 bp (AZFb)	sY134:301bp(AZFb)
sY254: 400 bp (AZFc)	sY255:126bp (AZFc)

**Table 2:** Sequence of PCR primers, multiplex A and B

	**Multiplex Primers**
**A and B**	**SRY-F: **5′-GAA TAT TCC CGC TCT CCG GA-3′
	**SRY-R: **5′-GCT GGT GCT CCA TTC TTG AG-3′
**A.**	**sY86-F: **5′-GTG ACA CAC AGA CTA TGC TTC-3′
	**sY86-R: **5'-ACA CAC AGA GGG ACA ACC CT-3′
**A.**	**sY127-F: **5ʹ-GGC TCA CAA ACG AAA AGA AA-3ʹ
	**sY127-R: **5'-CTG CAG GCA GTA ATA AGG GA-3ʹ
**A.**	**sY254-F: **5ʹ-GGG TGT TAC CAG AAG GCA AA-3ʹ
	**sY254-R: **5ʹ-GAA CCG TAT CTA CCA AAG CAG C-3ʹ
	**sY84-F: **5ʹ-AGA AGG GTC TGA AAG CAG GT-3ʹ
**B.**	**sY84-R: **5ʹ-GCC TAC TAC CTG GAG GCT TC-3ʹ
	**sY134-F: **5ʹ-GTC TGC CTC ACC ATA AAA CG-3ʹ
**B.**	**sY134-R: **5ʹ-ACC ACT GCC AAA ACT TTC AA-3ʹ
	**sY255-F: **5ʹ- GTT ACA GGA TTC GGC GTG AT – 3ʹ
**B.**	**sY255-R: **5ʹ - CTC GTC ATG TGC AGC CAC- 3ʹ

**Figure 1:**
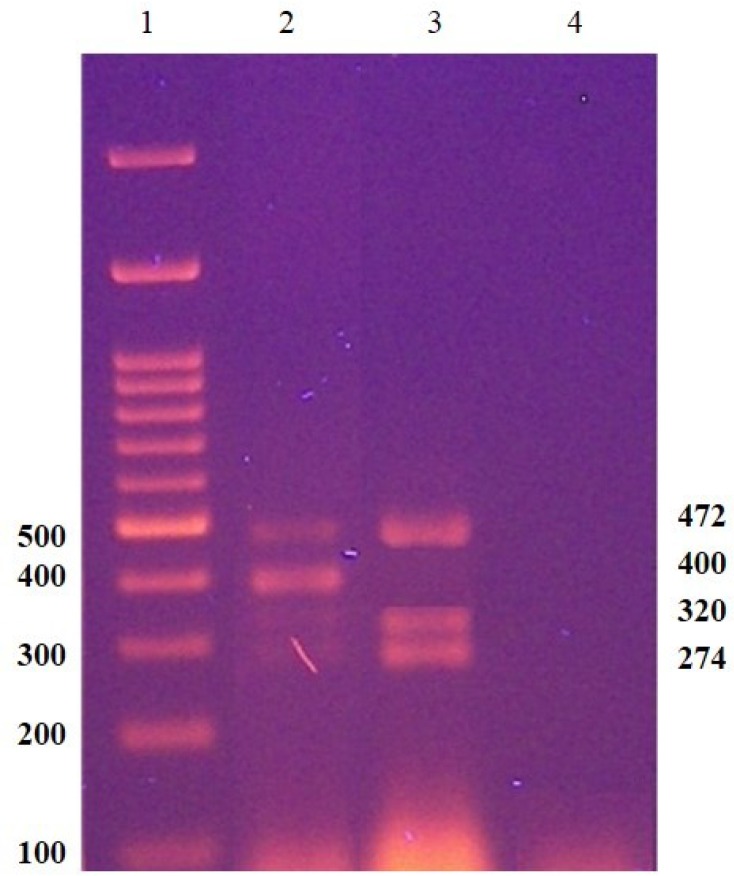
A representative of multiplex A PCR results on agarose gel, lane 1: Marker. Lane 2: DNA of normal male. Lane 3: DNA of a patient with AZFc complete microdeletion (absence of sY 254, 400bp), Lane 4: Water

**Figure 2:**
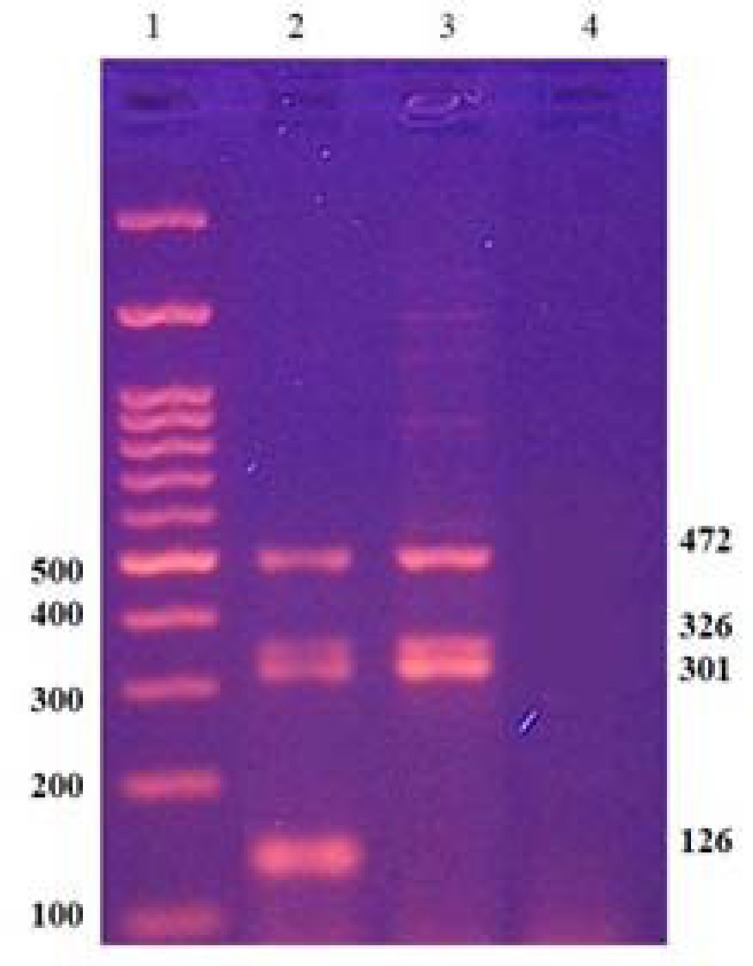
A representative of multiplex B PCR results on agarose gel, lane 1: Marker. Lane 2: DNA of normal male. Lane 3: DNA of a patient with AZFc complete microdeletion (absence of sY 255, 126bp), Lane 4: Water

Absence of sY 254 marker, 400 bp band, in multiplex A (Fig. 1) and absence of sY 255 marker, 126bp band, in multiplex B (Fig. 2) represent AZFc complete deletion. Robust and reproducible results have been obtained from these STS markers by many laboratories [3]. sY14 (SRY) was applied as a positive control for the testis-determining factor located on the short arm of Y chromosome. A fertile male and a female sample were also served as normal controls. A blank sample was applied in PCR reactions as a negative control. Both sets of multiplex PCR were carried out using Amplicon Multiplex PCR Master Mix (containing HotStart Taq DNA Polymerase, multiplex buffer with 1.5mM MgCl2 and dNTP mix). PCR conditions were as follows: initial activation for 15 min at 94°C, followed by 35 cycles of 60 sec denaturation at 94°C, 35 sec annealing at 58.5°C, and 30 sec extension at 72°C, and 1 cycle of final extension at 72°C for 10 min.

PCR products were run on 1.5% and 2% agarose gel for multiplex A and B, respectively. Ethidium bromide was used to visualize the bands by UVtransilluminator.

**Figure 3:**
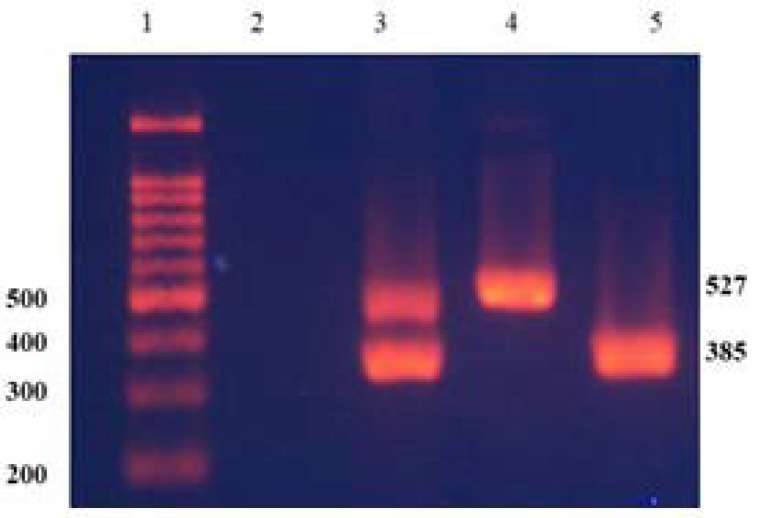
A representative of multiplex PCR results on agarose gel for AZFc partial microdeletions of the Y chromosome, lane 1: Marker. Lane 2: Water, Lane 3: DNA of normal male. Lane 4: DNA of a patient with b2/b3 microdeletion (absence of sY 1191, 385bp), Lane 5: DNA of a patient with gr/gr microdeletion (absence of sY 1291, 527 bp

**Analysis of partial microdeletions: **To determine the gr/gr subdeletions, two STS primers, sY1291 and sY1191, were applied in a multiplex PCR reaction. Both primers were chosen according to the EAA/EMNQ suggestion [3] to detect the gr/gr deletion (Table 3). The following PCR condition was applied: Initial activation for 15 min at 94°C, followed by 30 cycles of 60 sec of denaturation at 94°C, 35 sec of annealing at 59.5°C and 30 sec of extension at 72°C, and a final extension at 72°C for 10 min. PCR products were run on 1% agarose gel. Ethidium bromide was used to visualize the bands by UV transilluminator. The expected product size for sY1191 and sY1291 was 385 and 527bp, respectively. Presence of sY1191 and absence of sY1291 confirms gr/gr deletions while the absence of sY1191 and presence of sY1291 represent another partial microdeletion called b2/b3 (Fig. 3).

**Table 3:** Sequence of PCR primers to detect partial microdeletions (gr/gr and b2/b3

**Primers**	**Product size**
sY1291-F 5′-TAA AAG GCA GAA CTG CCA GG-3′	527bp
sY1291-R 5′-GGG AGA AAA GTT CTG CAA CG-3′	
	
sY1191-F 5′-CCA GAC GTT CTA CCC TTT CG-3′	385bp
sY1191-R 5′-GAG CCG AGA TCC AGT TAC CA-3′	

**Statistical analysis: **Statistical analysis was performed using SPSS, the Statistical Package for Social Sciences, (version 23; IBM corp., USA). The Chi square test was applied in order to detect any significant difference between patient and control groups in terms of carrying either complete or partial microdeletion. P value < 0.05 was considered statistically significant.

## RESULTS AND DISCUSSION

No AZFa or AZFb complete microdeletion was detected in any of patients in this study. One azoospermic male out of 81 infertile males (1.2%) had complete deletion of AZFc (Table 4). There was no significant difference (p=0.61) between patient and control groups regarding carrying complete microdeletion in the Y chromosome. In 1976, Tiepolo and Zuffardi showed that Y chromosome microdeletions could cause spermatogenic failure [21]. Since then, many studies have investigated the frequency of these microdeletions. Depending on the patients selection criteria, genetic background and ethnicity, the frequency of these microdeletions may vary considerably. Moreover, lack of consistency in protocols applied to detect microdeletions and methodological errors may lead to variation in the results [3, 15, 16, 20]. In Iran, there are reports of Y chromosome microdeletions frequency which are markedly higher compared to other countries. Malekasgar and Mombaini showed 51.6% of azoospermic infertile men and 52.6% of severe oligozoospermic patients from North of Iran carrying Y chromosome microdeletions [17]. Omrani et al., reported 24% microdeletions in infertile men from West Azarbayjan [22]. Some of Iranian studies had methodological errors [15, 16] and some applied many STS markers while there are standardized STS markers endorsed by the EAA and EMQN which can detect 95% of all reported AZF microdeletions [20]. In the current study, we followed EAA/EMQN procedure and detect only 1.25% complete microdeletions in our patients. It is noteworthy to mention that optimizing the PCR conditions and applying confirmatory steps such as simplex PCR is necessary as we observed higher frequency of microdeletions at first. Our data is consistent with the report by Saliminejad et al., in 2012, which showed a frequency of 2.13% of complete microdeletions in the Y chromosome in an Iranian population [18].

**Table 4:** Patients with either partial or complete microdeletion in the Y chromosome

	**Multiplex A**				**Multiplex B**				**Partial AZFc deletion**	
**Patient Number**	**sY14:**	**sY254:**	**sY86:**	**sY127**	**sY14:**	**sY84:**	**sY134:**	**SY255:**	**sY1291**	**sY1191**
	**SRY**	**AZFc**	**AZFa**	**:AZFb**	**SRY**	**AZFa**	**AZFb**	**AZFc**		
**#6 (Azoospermic)**	**+**	**-**	**+**	**+**	**+**	**+**	**+**	**-**	**-**	**-**
**#10 (Azoospermic)**	**+**	**+**	**+**	**+**	**+**	**+**	**+**	**+**	**-**	**+**
**#11 (Azoospermic)**	**+**	**+**	**+**	**+**	**+**	**+**	**+**	**+**	**-**	**+**
**#39 (Azoospermic)**	**+**	**+**	**+**	**+**	**+**	**+**	**+**	**+**	**-**	**+**
**#53(Oligospermic)**	**+**	**+**	**+**	**+**	**+**	**+**	**+**	**+**	**-**	**+**
**#73(Oligospermic)**	**+**	**+**	**+**	**+**	**+**	**+**	**+**	**+**	**+**	**-**

After excluding the patient with complete deletion of AZFc, presence of partial microdeletion of this region was investigated. In total, 6.25% (5 out of 80) of infertile males showed AZFc partial microdeletions including 4 gr/gr (5%) and one b2/b3 subdeletion (1.25%) (Table 4). 2 out of 50 controls (4%) including normospermic and/or proven fathers showed gr/gr deletions while no b2/b3 deletion was detected in controls (Table 5). There was no significant difference (p=0.45) between patient and control groups regarding carrying partial microdeletion in the Y chromosome.

Susceptibility of AZFc region to non-homologous recombination leads to both partial deletions and duplications which changes the gene dosage [reviewed in 3]. In 2014, EAA/EMQN has published another guide line in which detection of a partial deletion of AZFc called gr/gr was suggested for infertile men with spermatogenic failure [3]. However, in some Asians with specific Y haplogroup, this microdeletion is fixed without affecting the spermatogenesis [23, 24]. Here, we have shown that there are 5% gr/gr

microdeletions in our patients. However, we have also detected gr/gr microdeletions in 4% of our control group, which was a mixture of proven fathers and/or normospermic individuals. These results suggest that in our study group, gr/gr microdeletions may not be involved in spermatogenic failure. However, Motevali-Bashi et al., recently showed that there is a higher frequency of gr/gr microdeletions in azoospermic and severe oligozoospermic patients attended to the Isfahan infertility center compared to control group [25]. Difference in patient selection criteria or sample size may lead to these controversial results. Another partial deletion, b2/b3 was detected in our patients with a frequency of 1.25 % while no b2/b3 deletions was detected in our control group. Interestingly, Eloualid et al, reported the same frequency (1.22%) for b2/b3 microdeletions in their infertile patients [26]. While b2/b3 microdeletions shows no association with spermatogenic failure in some populations with specific Y chromosome background [27], it is associated with male infertility in a Chinese population [28].

**Table 5:** Controls with partial microdeletion in the Y chromosome

		**Multiplex A**				**Multiplex B**				**Partial AZFc deletion**	
**Control Number**	**sY14:**		**sY254:**	**sY86:**	**sY127**	**sY14:**	**sY84:**	**sY134:**	**SY255:**	**sY1291**	**sY1191**
	**SRY**		**AZFc**	**AZFa**	**:AZFb**	**SRY**	**AZFa**	**AZFb**	**AZFc**		
**#5 (Proven father)**	**+**		**+**	**+**	**+**	**+**	**+**	**+**	**+**	**-**	**+**
**#12 (Normospermic**	**+**		**+**	**+**	**+**	**+**	**+**	**+**	**+**	**-**	**+**

In conclusion, current data shows that there is a low frequency of Y chromosome microdeletions, either complete or partial, in azoospermic and oligozoospermic infertile men in a population of the South of Iran. A partial microdeletion, b2/b3, was only found in patients and not in control group which may suggest the importance of screening for this microdeletion in infertile men of this population. However, we recommend a larger group of patients and controls to be screened for this microdeletion for confirmation.
